# A splicing variation in *NPRL2* causing familial focal epilepsy with variable foci: additional cases and literature review

**DOI:** 10.1038/s10038-021-00969-z

**Published:** 2021-08-11

**Authors:** Jia Zhang, Yajun Shen, Zuozhen Yang, Fan Yang, Yang Li, Bo Yu, Wanlin Chen, Jing Gan

**Affiliations:** 1 Department of Pediatrics, West China Second University Hospital, Sichuan University, Chengdu, China; 2 Key Laboratory of Obstetrics & Gynecologic and Pediatric Diseases and Birth Defects of the Ministry of Education, Sichuan University, Chengdu, Sichuan China; 3 Cipher Gene LLC, Beijing, China; 4 Department of Pediatrics, The City Central Hospital of Wanyuan, Wanyuan, Sichuan China

**Keywords:** Genetics research, Neurological disorders

## Abstract

NPRL2 (nitrogen permease regulator like 2) is a component of the GATOR1(GAP activity towards rags complex 1) proteins, which is an inhibitor of the amino acid-sensing branch of the mTORC1 pathway. GATOR1 complex variations were reported to correlate with familial focal epilepsy with variable foci (FFEVF). However, FFEVF caused by *NPRL2* variants has not been widely explored. Here, we describe a variant, 339+2T>C, in *NPRL2* identified by trio whole-exome sequencing (WES) in a family. This splicing variant that occurred at the 5′ end of exon 3 was confirmed by minigene assays, which affected alternative splicing and led to exon 3 skipping in *NPRL2*. Our cases presented multiple seizure types (febrile seizures, infantile spasms, focal seizures, or focal to generalized tonic-clonic seizures). Electroencephalogram (EEG) showed frequent discharges in the left frontal and central regions. A favorable prognosis was achieved in response to vitamin B6 and topiramate when the patient was seven months old. Our study expands the phenotype and genotype spectrum of FFEVF and provides solid diagnostic evidence for FFEVF.

## Introduction

NPRL2 is a component of the GATOR1 complex, along with nitrogen permease regulator like-3 (NPRL3) and DEPDC5. The NPRL2 protein is expressed in variable regions of the human brain, including the frontal, temporal, parietal, and occipital lobes, similar to *DEPDC5* [1]. NPRL2 is also known as tumor suppressor candidate 4 [2], which is upregulated in primary prostate cancer tissues [3]. The GATOR1 complex inhibits mechanistic target of rapamycin (mTOR) activation according to the amino acid levels in ambient cells [4]. It changes the nucleotide loading status (GTP or GDP) of the Rag proteins and deactivates them to release mTOR complex 1 (mTORC1) from the lysosome [5, 6].

mTORC1 has been described as a possible cause of epileptogenesis, and increased activity participates in seizure progression [7, 8]. Overall, germline variants in the GATOR1 complex genes (*DEPDC5*, *NPRL3*, and *NPRL2*) are present in ~10% of focal epilepsy cases [9], which can be familial or sporadic, especially in familial focal epilepsy with variable foci (FFEVF). Moreover, generalized epilepsy and infantile spasms have also been reported [10]. Loss-of-function variants in *DEPDC5* and *NPRL3* have been investigated extensively in both animal models and human tissues associated with mTORC1 hyperactivation [11, 12]. However, variants in *NPRL2* related to epileptogenesis are still not well understood.

Limited studies have suggested that variants of *NPRL2* are correlated with focal epilepsy, which may be associated with focal cortical dysplasia (FCD) and intellectual disability [1, 10, 13]. Our study uncovered a new variation (NM_006545.5, c.339+2T>C) in *NLPR2* in a 7-month-old infant with FFEVF that was inherited from his mother. The variation was confirmed to impact the alternative splicing of *NPRL2*, which may be the consequence of neurogenesis dysplasia through disturbing the mTOR signaling pathway. Our study expands the phenotype and genotype spectrum of *NPRL2*.

## Materials and methods

### Patient

Informed consent was obtained from the parents and their families. This study was approved by the institutional review board of the West China Second University Hospital. The patient’s clinical manifestations, electroencephalogram (EEG), brain magnetic resonance imaging (MRI), malformations, investigations of other organs, and gene variations were analyzed. We also combined the *NPRL2* variant-related cases reported previously in our analysis. Additional phenotype data and genetic findings for individuals are summarized in Table 1.

**Table 1:** NPRL2 variations and associated phenotypes in individuals with focal epilepsy identified to the present.

Case No.	Variation No.	Reference allele	Alternative allele	cDNA variant	Protein alteration	Variant class	GnomAD allele count	Novel classification	Gender	Age onset	Epilepsy phenotype	EEG	MRI	Neuropsychiatric comorbidities	SUDEP in the family	Familial /Sporadic	Inheritance	Penetrance	Reference	PIMD
1	1	G	C	c.1134C>G	p.Cys378Trp	Missense	0	*VUS*	Male	3y 8m	Frontal lobe epilepsy	Interictal: right frontal epileptiform activity. Ictal (subclinical): right fronto-central sharp waves, activated by sleep	FCD (left parieto-temporal)	N/A	No	N/A	N/A	N/A	Baldassari et al. [10]	30093711
2	2	G	A	c.883C>T	p.Arg295*	LoF	0	Pathogenic	N/A	N/A	Temporal lobe epilepsy	N/A	N/A	No	No	Familial	N/A	Incomplete	Ricos et al. [1]	26505888
3	3	C	G	c.683+1G>C	p.(?)	LoF	0	Pathogenic	Female	<1w	Infantile spasms	Interictal: multifocal epileptiform abnormalities with left predominance	FCD (left parieto-temporal)	Intellectual disability	No	Sporadic	N/A	N/A	Baldassari et al. [10]	30093711
4	4	C	G	c.640G>C	p.Asp214His	Missense	22	*VUS*	N/A	N/A	Frontal lobe epilepsy, tumor-like brain lesion	N/A	N/A	No	No	Sporadic	N/A	N/A	Ricos et al. [1]	26505888
5	5	G	C	c.329C>G	p.Thr110Ser	Missense	0	*VUS*	N/A	N/A	Temporal lobe epilepsy, polymicrogyria	N/A	N/A	Intellectual disability	No	Sporadic	Inherited	Incomplete	Ricos et al. [1]	26505888
6	6	A	G	c.314T>C	p.Leu105Pro	Missense	0	Likely pathogenic	N/A	N/A	Sleep-related hypermotor epilepsy	N/A	N/A	No	No	Familial	Inherited	Incomplete	Ricos et al. [1]	26505888
7	6	A	G	c.314T>C	p.Leu105Pro	Missense	N/A	Likely pathogenic	N/A	N/A	Sleep-related hypermotor epilepsy	N/A	N/A	N/A	N/A	N/A	Inherited	N/A	Licchetta et al. [23]	31835056
8	7	G	A	c.232C>T	p.Arg78Cys	Missense	0	Likely pathogenic	Female	18y	Left temporal lobe epilepsy	Left temporal slow and spike waves	normal	No	No	Familial	Inherited	Incomplete	Perucca et al. [24]	28199897
9	8	G	A	c.100C>T	p.Arg34*	LoF	0	Pathogenic	N/A	N/A	Sleep-related hypermotor epilepsy	N/A	N/A	No	No	Familial	N/A	Complete	Ricos et al. [1]	26505888
10	8	G	A	c.100C>T	p.Arg34*	LoF	0	Pathogenic	Female	11m	Sleep-related hypermotor epilepsy	Interictal: discharges in right cingulate gyrus	FCD (right frontal)	Intellectual disability	No	Familial	Inherited	N/A	Baldassari et al. [10]	30093711
11	9	TGA	T	c.68_69delTC	p.Ile23Asnfs*6	LoF	N/A	Pathogenic	Female	3y	Frontal lobe epilepsy	SEEG: right frontoinsular and frontoorbital onset	Normal	N/A	N/A	Familial	Inherited	N/A	Weckhuysen et al. [13]	27173016
12	9	TGA	T	c.68_69delTC	p.Ile23Asnfs*6	LoF	0	Pathogenic	Female	13y	Temporal lobe epilepsy	Left temporal discharges	Normal	No	Yes	Familial	Inherited	Incomplete	Weckhuysen et al. [13]	27173016
13	10	C	T	c.562C>T	p.Gln188*	LoF/Nonsense	N/A	Pathogenic	Male	2y	N/A	N/A	Left superior frontal gyrus FCD	N/A	N/A	N/A	Inherited	N/A	Alissa et al. [25]	29281825
14	11	G	A	c289G>A,	p. Ala97Thr	Missense	N/A	*VUS*	Male	8m	Temporal lobe epilepsy	N/A	N/A	No	N/A	Familial	Inherited	N/A	Deng et al. [26]	31594065
15	12	T	C	c.399+2T>C	p.(?)	Splicing	0	Likely pathogenic	Male	4d	focal onset or focal to generalized tonic-clonic seizures; Spastic Seizures	Frequent discharges in left frontal and central regions	Hypoperfusion in the cortex of the left frontal and parietal regions	Moderate developmental delay	No	Familial	Inherited	Complete	Proband in our study	our case
16	12	T	C	c.399+2T>C	p.(?)	Splicing	0	Likely pathogenic	Female	2y	Febrile Seizures	Occasional discharges in the left frontal regions	Relatively hypoperfusion in the left frontal and temporal lobes	Intellectual disability	No	Familial	Inherited	Complete	Proband’s mother in our study	our case

### Whole-exome sequencing (WES) and Sanger sequencing

To further clarify the patient’s diagnosis, genomic DNA was extracted from the peripheral blood of the patient and his parents. WES was performed based on the NovaSeq 6000 Sequencing platform, IDT XGen Exome Research Panel was used to capture libraries, and paired-end clean reads were used to compare to the human reference genome (GRCh38/hg38). Variations were annotated through ANNOVAR [14] and picked up with a minor allele frequency of ≤0.005 in the SNP database.

WES uncovered potential pathogenic variants. All variants were evaluated according to the American College of Medical Genetics and Genomics (ACMG) guidelines. “Ada” and “RF” scores were used to evaluate potential splicing variants predicted by dbscSNV. Sanger sequencing was performed to validate the variation identified by WES.

### Minigene construction

Minigene assays were performed to investigate *NPRL2* splicing via WT genomic DNA amplification. The construction contained exon 2–exon 3–exon 4 in *NPRL2*. Nested PCR was performed to amplify the targeted DNA fragment through normal peripheral blood. Amplification products were successfully cloned and confirmed by Sanger sequencing. Variation c.339+2T>C in *NPRL2* was constructed by site-directed mutagenesis. Both wild-type and mutant fragments were delivered into the pcDNA3.1 vector after digestion and connection. All primers used in the minigene construct are provided in Supplementary Table 1.

### Cell transfection

HeLa and 293 cells were cultured in DMEM supplemented with 10% fetal bovine serum. These minigenes, which are named pcDNA3.1-*NPRL2*-WT/MUT, were transfected into 293T cells using Lipo2000 Transfection Reagent (11668019, 205 Invitrogen) according to the manufacturer’s protocol. The DNA-lipid complex was incubated for 15 min in Opti MEM medium (Gibco, Grand Island, NY) at room temperature before addition to the cells.

### RT-PCR

Total RNA was extracted 48 h after transfection using RNAiso PLUS (9109, TaKaRa). Retrotranscription was performed using a Prime Script RT Reagent kit with gDNA Eraser (RR047A, TaKaRa). Primers are shown in Supplementary Table 1. PCR was performed and evaluated on a 1% agarose gel. Subsequently, potential changes in the splicing process were identified by direct sequencing.

### 3D protein structure modeling

Molecular modeling analysis was performed to estimate the variant in protein structure. WT and variations in NPRL2 protein were predicted using the Swiss-Model program. Swiss-Pdb Viewer software was used to visualize the structures between WT and variation proteins.

## Results

### Case presentation

A 7-month-old male infant was born after an uneventful full-term pregnancy. He had multiple unprovoked seizures 4 days after birth, predominantly focal seizures or focal to generalized tonic–clonic seizures in semiology. The seizures occurred six times a day, most of which lasted 10 s and exhibited no diurnal differences. He developed spastic seizures when he was 1 month old. He was unable to roll over upon admission at 4 months of age with generalized hypotonia evident on the right side. There were no neurocutaneous markers, specific facial features, or any other systemic abnormalities. His occipitofrontal head circumference was 42.5 cm. A diagnosis of developmental epileptic encephalopathy was considered. Interictal EEG demonstrated frequent sharp and slow waves in the left frontal and central regions (Supplementary Fig. 2a). Ictal EEG demonstrated the typical findings of epileptic spasms (Supplementary Fig. 2b–d). Brain CT, blood glucose, and electrolyte levels were normal. There were no abnormalities in the initial screening for infection or in the blood and urine metabolic screening. In addition, the auditory brainstem response and visual evoked potential tests were negative. His seizures were effectively controlled by a large dose of vitamin B6 (50 mg/kg/d*2 weeks) and topiramate (8 mg/kg/d). Since the seizures free were acquired, the parents refused the treatment of adrenocorticotropic hormone. However, he still exhibited a failure to thrive, and his development was delayed and stagnant. At his latest follow-up at 7 months of age, he was seizure-free and sustained on a combination of topiramate (8 mg/kg/d) and vitamin B6 (10 mg bid) therapy. He had moderate developmental delay, with a Gesell Developmental Scale score of 45. He could lift his neck at 3 months of age and roll over at 5 months of age. However, he will not sit alone or take the initiative to grasp at 7 months old. Repeated EEG monitoring showed improvement in epileptiform discharge with intermittent sharp and slow waves in the left frontal and central regions. Head MRI showed no obvious signal abnormalities (Supplementary Fig. 1a–c). Interictal arterial spin labeling (ASL)-MRI showed relative hypoperfusion in the cortex of the left frontal and parietal regions (Supplementary Fig. 1g–j). His mother had an intellectual disability (WISC Scale score of 53). Her head MRI was normal (Supplementary Fig. 1d–f), while her ASL-MRI showed relative hypoperfusion in the left frontal and temporal lobes (Supplementary Fig. 1k–n). Her EEG showed occasional sharp and slow waves in the left frontal regions (Supplementary Fig. 2e, f). Both the patient’s mother and grandfather had a history of febrile seizures in childhood.

### Identification of *NPRL2* variation related to family focal epilepsy with variable foci

**Fig. 1:**
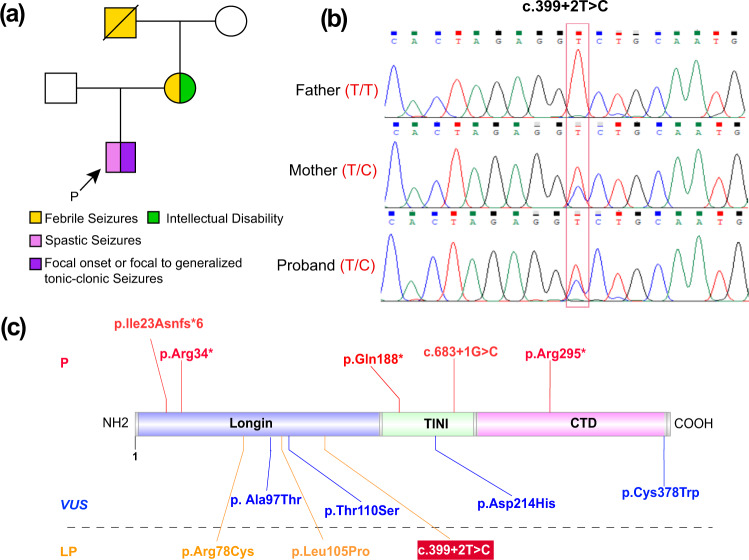
**Identification of a heterozygous variation in NPRL2**. **a** Pedigree of the family. The proband affected with focal epilepsy is indicated by filled symbols with arrows. **b** Sanger sequencing of the proband and his parents showed a c.339+2T>C mutation (red translucent box) in the *NPRL2* gene, which was inherited from the mother. **c** Domain structure and modeling of *NPRL2* variations in previous studies. The upper panel indicates the pathogenic (P) variants (red font). Variants of uncertain significance (*VUS*) and likely pathogenic (LP) are presented below. The NPRL2 protein contains an N-terminal longin domain (NLD), a tiny intermediary of *NPRL2* that interacts with the *DEPDC5* domain (TINI), and a C-terminal domain (CTD). The variation in our study in the red box was LP.

WES was performed to further clarify the diagnosis and explore the etiology of our patient. A heterozygous variant (NM_006545.5: exon 3: c.339+2T>C) in the *NPRL2* gene was uncovered, and it was inherited from his mother (heterozygous) as determined through Sanger sequencing (Fig. 1a, b). The variant was a canonical splicing site in the 5′ end of intron 3, which may impact alternative splicing.

Neither the GnomAD nor Exome Aggregation Consortium (ExAC) databases exhibited this variation, indicating the rarity of this mutation. It was also predicted to be splicing-influencing by “Ada” and “RF” scores (Table 2). Only 27 variants of *NPRL2* were included in the ClinVar database (https://www.ncbi.nlm.nih.gov/clinvar/?term=NPRL2%5Bgene%5D), 16 of which were pathogenic or likely pathogenic. To date, 11 variants in the *NPRL2* gene have been described in 14 epilepsy probands (Fig. 1c, Table 1). Fifty percent (7/14) of them are pathogenic, 21.4% (3/14) are likely pathogenic, and 28.6% (4/14) are variants of unknown significance (*VUS*). Five variants have been reported to have loss of function (LoF) and were classified as pathogenic in unrelated cases. The c.339+2T>C mutation in our study was also designated as likely pathogenic by the ACMG guidelines.

**Table 2:** Clinical examinations and variant information.

Gene	Mutation	Inheritance	MAF			dbscSNV_ AD_SCORE	dbscSNV_ RF_SCORE	Category
			ExAc	GnomAD	1000 Genome			
*NPRL2*	c.339+2T>C	Inherited	NE	NE	NE	0.9995	0.866	LP

Transcript: NM_006545.5.

*MAF* minor allele frequency, *NE* not exist, *LP* likely pathogenic.

### Functional splicing examination of the variant through minigene assays

**Fig. 2:**
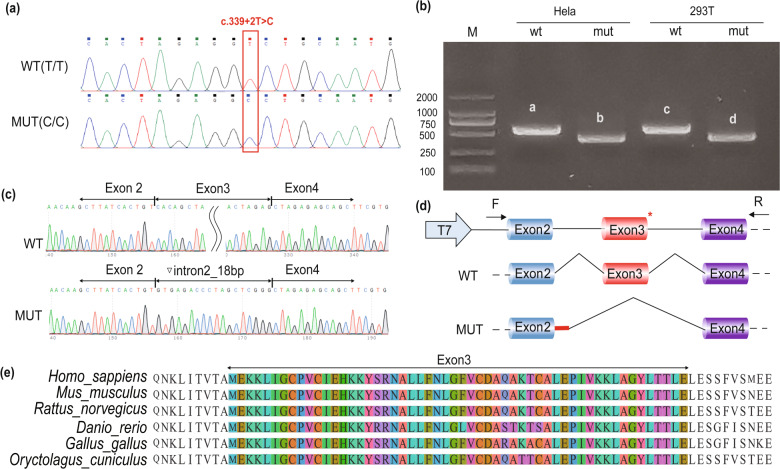
**Minigene assays and identification of the variant’s impact on alternative splicing**. **a** Sanger sequencing confirmed that wild-type and mutant fragments were successfully introduced into the minigene construct. Splicing variation c.339+2T>C in *NPRL2* is indicated by the red box. **b** RT-PCR was performed to verify alternative splicing in the wild-type and mutant groups. Abnormal splicing bands in mutant groups, named “b” and “d”, were uncovered in both HeLa and 293 T cells. At the same time, normal bands, named “a” and “c”, were indicated in the wild-type group. **c** Alternative splicing was affected by the c.339+2T>C variation in *NPRL2*. PCR product sequencing revealed 18 bp intron 2 retention and exon 3 skipping. The alternative schematic is shown in (**d**). The red “*” symbolizes the variation site. Intron 2 retention is indicated in the mutant group by a red line. **e** Species conservation analysis of exon 3 in *NPRL2*.

Since the c.339+2T>C variant changed the 5′ donor site ‘gt’ in the intron region, a splicing assay was performed to further confirm the influence of alternative splicing. Target DNA fragments were successfully inserted into the pcDNA3.1 vector and confirmed by Sanger sequencing (Fig. 2a). RT-PCR results indicated two different splicing patterns, named “a” and “c” and “b” and “d” (Fig. 2b). Sanger sequencing uncovered abnormal splicing in products “b” and “d”. Intron 2 retention and exon 3 skipping of 18 bp were observed in the mutant group (Fig. 2c, d). The schematic for abnormal splicing is shown in Fig. 2d. The red asterisk indicates the variation site. In addition, exon 3 in *NPRL2* is highly conserved across multiple species (Fig. 2e).

### Protein modeling

**Fig. 3:**
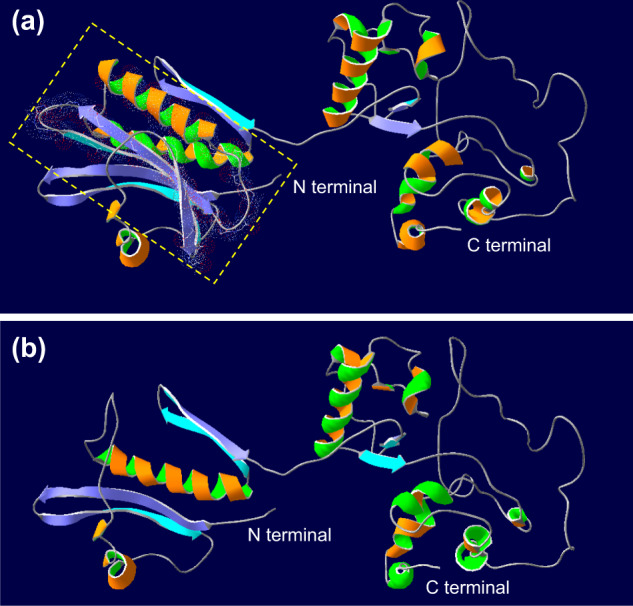
**Protein 3D structures in WT and the NPRL2 variant**. **a** WT protein. **b** Variant protein. The yellow dotted box indicates the different regions of the WT NPRL2 protein compared to the variation.

Swiss-Pdb Viewer was used to predict and compare the structures of NPRL2 in the WT and variant. Variation 339+2T>C causes exon 3 skipping, which results in several amino acid stretches being deleted. Two sheets and one helix for NPRL2 in the N-terminal region disappeared compared with the WT (Fig. 3). These regions were highlighted with amino acid residues and van der Waals dots and will significantly affect the function and stability of the NPRL2 protein.

## Discussion

mTORC1 is well known to be involved in cell differentiation and protein synthesis and is highly expressed in the brain and regulates neurogenesis. Defects in the mTOR pathway play an important role in focal epilepsy during brain development. GATOR1, a negative regulator of mTORC1, consists of components DEPDC5, NPRL2, and NPRL3. In recent years, GATOR1-related variations have been gradually recognized in epilepsy patients via the wide application of second-generation sequencing, causing ~10% of focal epilepsy [1, 13]. GATOR1 variations that activate the mTORC1 pathway assume the main responsibility for focal epilepsy with cortical malformations, representing a potential target for novel therapeutics [10].

Familial focal epilepsy (FFE) is characterized as a genetically distinct type of epilepsy syndrome, primarily including autosomal dominant nocturnal frontal lobe epilepsy, familial medial temporal lobe epilepsy, familial lateral temporal lobe epilepsy, and FFEVF, which are considered to primarily result from ion channel and neurotransmitter receptor gene variations. FFEVF is a typical subtype that has been reported to be primarily associated with *DEPDC5* variations. At the same time, ~12% of FFE patients have been found possess *DEPDC5* variations [15]. Epilepsy syndrome caused by *NPRL2* and *NPLR3* variations is similar to *DEPDC5* [1]. EEG showed that the initial discharge site was changeable, mostly from the frontal lobe or temporal lobe. However, no obvious correlation between EEG discharge and clinical manifestations was found [16]. GATOR1-related FFEVF patients always have a family history, with incomplete penetrance and heterogeneous clinical manifestations. Some of them exhibit focal EEG discharge without clinical symptoms. Most patients present with normal psychomotor development, while some may have mild cognitive decline or autism-like manifestations without seizures [1, 15]. Similarly, our case presented multiple seizure types, including infantile spasms, focal seizures, or focal to generalized tonic-clonic seizures. Both his mother and grandfather had a history of febrile seizures when they were young. In addition, his mother had an intellectual disability.

Approximately 20% of patients with GATOR1-related focal epilepsy have FCD [10, 13, 16]. In our study, no obvious abnormalities were observed in MRI of this proband. ASL-MRI showed relative hypoperfusion in the left frontal and parietal lobes, consistent with the epileptiform discharge detected by video EEG. It has been reported that 55–78% of GATOR1-related focal epilepsy patients are drug-resistant [10, 17]. Patients with definite lesions had better surgical results, with a 50–60% complete remission rate [10, 18]. Fortunately, our case had a favorable prognosis with the combination treatment of topiramate and vitamin B6, and the video EEG on follow-up showed a significant decrease in epileptiform discharges. Considering the focal seizures with epileptic spasms of the proband, high-dose vitamin B6 and topiramate were applied according to the guidelines and consensus on the treatment of infantile spasms and epileptic encephalopathy [19]. However, there are not any apparent relationships between the *NPRL2* gene and the mechanisms of action of anti-seizure medications which needs more investigation in the future.

There is no clear correlation between genotype and phenotype in GATOR1 variations, with an incomplete penetrance from 50% to 82% [1, 15]. This likely results from a combination of genetic, environmental, and lifestyle factors [20]. Additionally, this may be due to the additive effect of multiple independent variations, which can disturb and often increase the severity of the phenotype. GATOR1-related epilepsies caused by *DEPDC5*, *NPRL2*, and *NPRL3* variations exhibit various phenotypes consisting of sleep-related focal hypermotor seizures, infantile spasms, and other focal epilepsies, including frontal, temporal, occipital, parietal, centrotemporal epilepsies [10]. The various phenotypes are the same as those of patients who have *NPRL2* variations. Previous studies also revealed distinct phenotypes in a family with the same variant [1, 13]. A single pathogenic variant could lead to variable phenotypic manifestations, including different age at onset, seizure type, seizure severity, drug response, and presence of cortical malformations [16], which was observed in our comparison (Table 1). The types of variations seem to have no connection with the epilepsy phenotypes, neuropsychiatric comorbidities, or abnormal brain structure. Interfamilial variability in genetic epilepsies is a common phenomenon. Therefore, the child presented with developmental epileptic encephalopathy with focal as well as spastic seizures, while the mother showed only mild mental retardation. The maternal grandfather had a history of febrile seizures at a young age, and then had no epileptic seizures, and the psychomotor development was normal. One explanation for how a single variant in the same family causes mild focal epilepsy or refractory epilepsy may be the occurrence of a second somatic mutation [18].

Haploinsufficiency has been shown to be the pathogenic mechanism in *NPRL2/3* variations with incomplete penetrance [13]. However, whether missense and splicing variations have clinical significance is still unclear. Functional evidence and strong segregation support were absent. Therefore, no missense or splicing variations were classified as pathogenic through the new framework of epilepsy-related GATOR1 classification [10]. Alternative splicing was confirmed by minigene assays, and exon 3 skipping was observed during transcription (Fig. 2). The mutation may destroy the original donor site in exon 3 and activate a cryptic splice site at the beginning of intron 2 [21, 22]. In addition, it should be mentioned that NPRL2 links DEPDC5 and NPRL3 to comprise the GATOR1 complex. Furthermore, NPRL2 interacts with DEPDC5 through its longin domain [6]. Variation in our study leads to exon 3 skipping located in the longin domain, which may affect the connection between NPRL2 and DEPDC5 and is critical for the function of the GATOR1 complex.

In summary, we describe an individual with multiple seizure types harboring a splicing variation in *NPRL2*. Exon 3 skipping was confirmed based on minigene assays, which may lead to a loss of function in NPRL2. Our study provides evidence for pathogenicity of the splicing variation in the GATOR1 complex and expands the phenotype and genotype spectrum of FFEVF, highlighting the critical role for *NPRL2* in neurodevelopment.

## Supplementary information

Supplementary materials

Supplementary fig 1

Supplementary fig 1
