# Supplementary material for: A splicing variation in NPRL2 causing familial focal epilepsy with variable foci: additional cases and literature review
Source: J Hum Genet. 2021 Aug 11;67(2):79–85. doi: 10.1038/s10038-021-00969-z (PMC8786660; doi:10.1038/s10038-021-00969-z)
Supplement: Supplementary file 1 — Supplementary materials [file 10038_2021_969_MOESM1_ESM.docx]

**Supplementary materials**

**Supplementary Fig 1.** Magnetic resonance imaging (MRI) (a-f) and Interictal ASL-MRI (g-n) results. Axial T1-weighted (a, d), T2-weighted (b, e), and fluid-attenuated inversion recovery (c, f) sequences showed no abnormal signals both in proband (a-c) and his mother (d-f). Interictal ASL-MRI of proband (g-j) showed hypoperfusion in the peripheral cortex of the left frontal parietal area (white arrows). Interictal ASL-MRI of mother (k-n) showed hypoperfusion in the left frontal and temporal lobes (white arrows).

**Supplementary Fig 2.** EEG results for the proband (a-d) and his mother (e-f). EEG (referential montage) on admission frequent sharp and slow waves in the left frontal and central regions (a). Ictal EEG demonstrated the typical findings of epileptic spasms (b-d). EEG (referential montage) demonstrated occasional sharp and slow waves in the left frontal region (e-f). [SENS *10 uv/mm, HF *70 Hz, TC *0.3 s, CAL*50 Hz].

**Supplementary Table 1.** Primers sequences in this study (Intron sequences showed in the minuscule).

| **assays** | **Primer name** | **sequences（5**’**- -3**’**）** |
| --- | --- | --- |
| Nest PCR for DNA fragments cloning | 261-*NPRL2*-F | ctgcctgaaggcagtagttt |
|  | 506-*NPRL2*-F | gcataggaagtttaggtaag |
|  | 1623-*NPRL2*-R | aggcccacaaaggggaagga |
|  | 1942-*NPRL2*-R | gccagccccaggtgatgata |
| Site-specific mutagenesis | *NPRL2*-MUT-F | GACCACACTAGAGgCctgcaatgagatggg |
|  | *NPRL2*-MUT-R | cccatctcattgcagGcCTCTAGTGTGGTC |
| Minigene construct | pcDNA3.1-*NPRL2*-KpnI-F | GCTTGGTACCGTCCCTGAAGACTTCATCTC |
|  | pcDNA3.1-*NPRL2*-BamHI-R | TAGTGGATCCATCAATGGGCAGAGTGCACCGG |
