# Supplementary figures and images for: A splicing variation in NPRL2 causing familial focal epilepsy with variable foci: additional cases and literature review
Source: J Hum Genet. 2021 Aug 11;67(2):79–85. doi: 10.1038/s10038-021-00969-z (PMC8786660; doi:10.1038/s10038-021-00969-z)

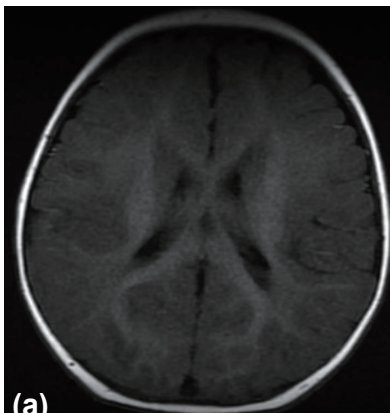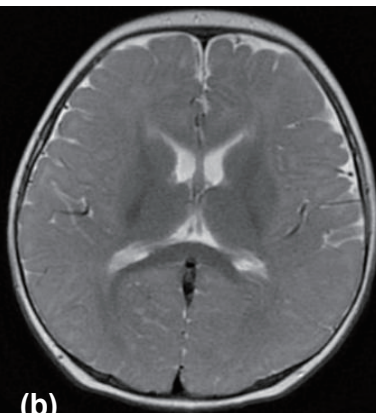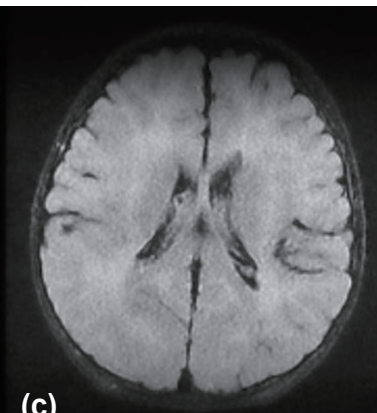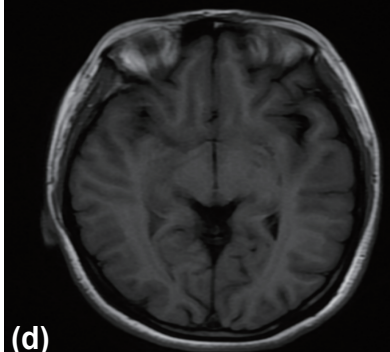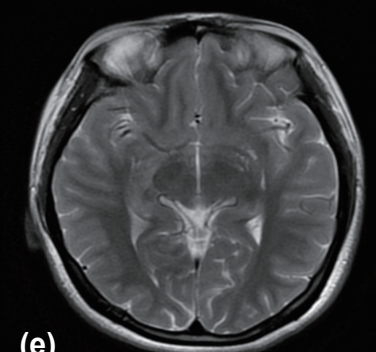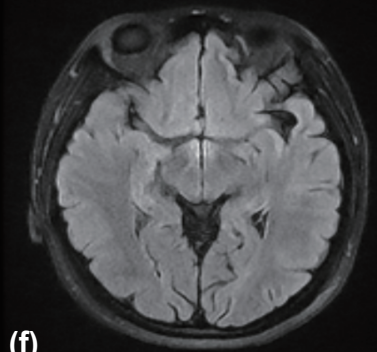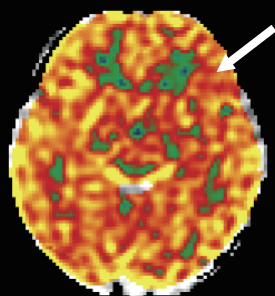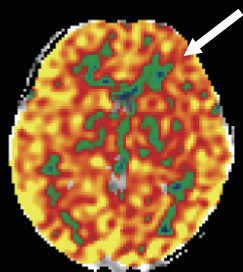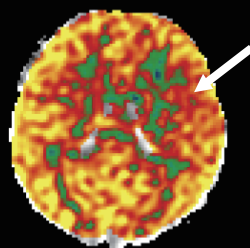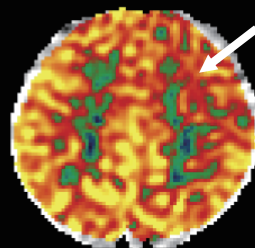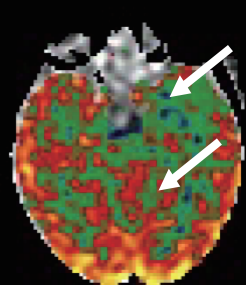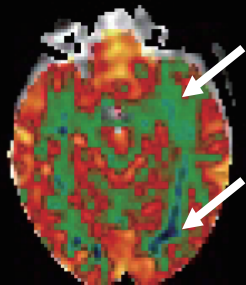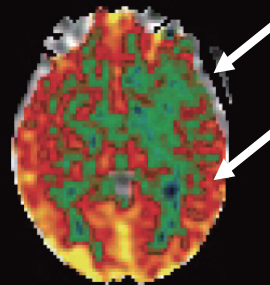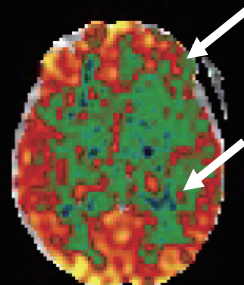

Supplement: Supplementary file 2 — Supplementary fig 1 [file 10038_2021_969_MOESM2_ESM.pdf]

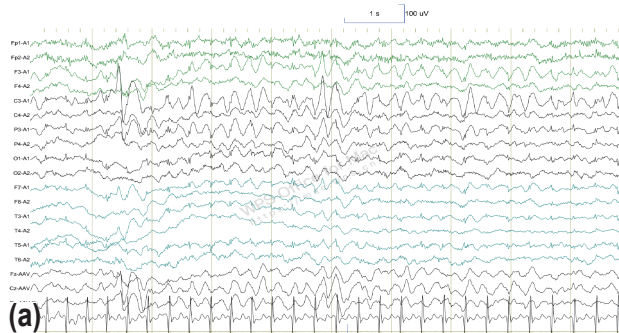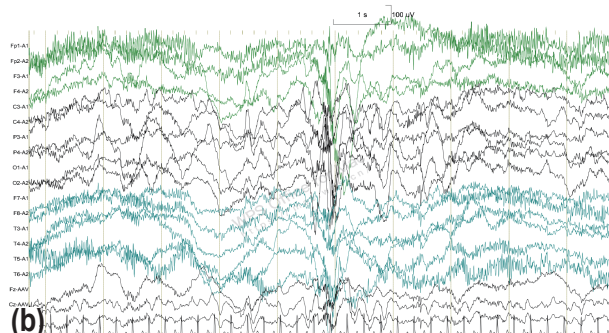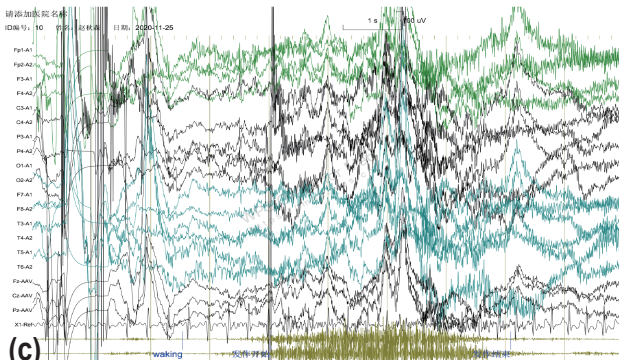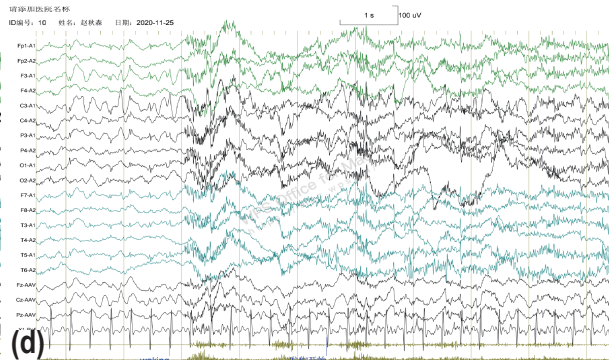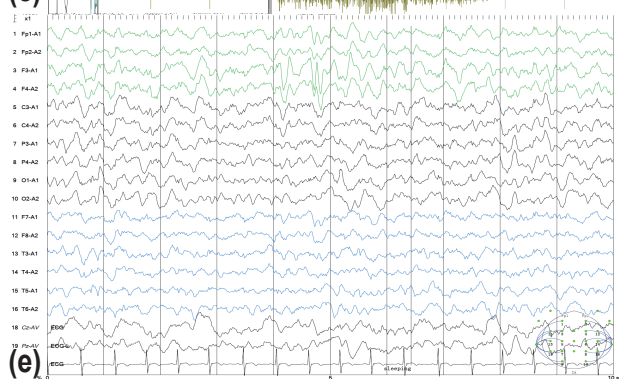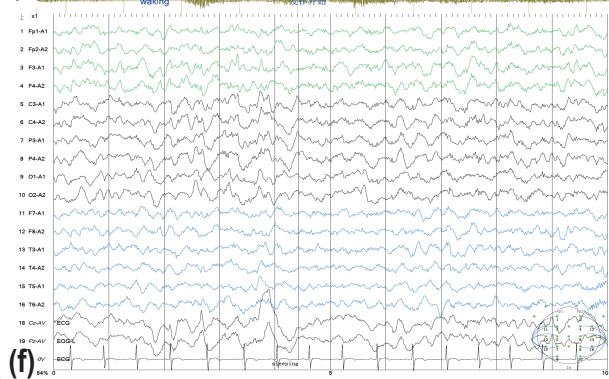

Supplement: Supplementary file 3 — Supplementary fig 1 [file 10038_2021_969_MOESM3_ESM.pdf]
